# Champions in context: which attributes matter for change efforts in healthcare?

**DOI:** 10.1186/s13012-020-01024-9

**Published:** 2020-08-06

**Authors:** Kirsten Bonawitz, Marisa Wetmore, Michele Heisler, Vanessa K. Dalton, Laura J. Damschroder, Jane Forman, Katie R. Allan, Michelle H. Moniz

**Affiliations:** 1 Department of Obstetrics and Gynecology, University of Michigan, 1500 E. Medical Center Dr, Ann Arbor, MI 48109 USA; 2 Department of Internal Medicine, University of Michigan Medical School, 300 North Ingalls, Ann Arbor, MI 48109 USA; 3 Institute for Healthcare Policy and Innovation, University of Michigan, 2800 Plymouth Rd., Building #10, Rm G016, Ann Arbor, MI 48109-5276 USA; 4 Veterans Affairs Center for Clinical Management Research, VA Ann Arbor Healthcare System, 2215 Fuller Rd, Ann Arbor, MI 48105 USA; 5 Geisel School of Medicine, Dartmouth, 1 Rope Ferry Rd, Hanover, NH 03755 USA

**Keywords:** Implementation champions, Quality improvement, Implementation science, Postpartum contraception, Maternity

## Abstract

**Background:**

Research to date has focused on strategies and resources used by effective champions of healthcare change efforts, rather than personal characteristics that contribute to their success. We sought to identify and describe champion attributes influencing outcomes of healthcare change efforts. To examine attributes of champions, we used postpartum contraceptive care as a case study, because recommended services are largely unavailable, and implementation requires significant effort.

**Methods:**

We conducted a comparative case study of the implementation of inpatient postpartum contraceptive care at 11 U.S. maternity hospitals in 2017–18. We conducted site visits that included semi-structured key informant interviews informed by the Consolidated Framework for Implementation Research (CFIR). Phase one analysis (qualitative content analysis using a priori CFIR codes and cross-case synthesis) showed that implementation leaders (“champions”) strongly influenced outcomes across sites. To understand champion effects, phase two inductive analysis included (1) identifying and elaborating key attributes of champions; (2) rating the presence or absence of each attribute in champions; and 3) cross-case synthesis to identify patterns among attributes, context, and implementation outcomes.

**Results:**

We completed semi-structured interviews with 78 clinicians, nurses, residents, pharmacy and revenue cycle staff, and hospital administrators. All identified champions were obstetrician-gynecologists. Six key attributes of champions emerged: influence, ownership, physical presence at the point of change, persuasiveness, grit, and participative leadership style. These attributes promoted success by enabling champions to overcome institutional siloing, build and leverage professional networks, create tension for change, cultivate a positive learning climate, optimize compatibility with existing workflow, and engage key stakeholders. Not all champion attributes were required for success, and having all attributes did not guarantee success.

**Conclusions:**

Effective champions appear to leverage six key attributes to facilitate healthcare change efforts. Prospective evaluations of the interactions among champion attributes, context, and outcomes may further elucidate how champions exert their effects.

Contributions to the Literature

- The importance of champions for healthcare change efforts is well established, but the existing literature has largely operationalized champions as a dichotomous state—did the initiative have a champion or not—and focused on champions’ strategies and resources.
- We provide rich qualitative data suggesting need to focus also on who a champion is. To what degree does a champion(s) have key attributes associated with effectiveness, and how well poised is a champion to address local barriers?
- Our findings advance the literature by newly identifying key attributes of champions and linking attributes to implementation outcomes, helping elucidate how and why champions work and potential mechanisms that can be evaluated in future prospective research.

## Background

Champions have long been regarded as key facilitators for successful change efforts in healthcare [1, 2]. Across hospital and outpatient settings, champions have facilitated change efforts by building organizational support for new practices, securing needed resources, and building organizational coalitions [3–9]. However, a recent integrative review found that the success of champions is variable [1]. Additionally, four recent studies randomly allocating the presence or absence of a champion found positive effects of champions on many, but not all outcomes [10–13].

Better understanding of who is leading implementation efforts—as well as how and why these leaders are effective, and in what contexts—can facilitate more successful efforts to identify and cultivate effective champions for change. Much of the existing literature focuses on the *strategies* and *resources* of effective leaders. Relatively little is known about the *attributes* of individuals who successfully lead implementation efforts. Further, many existing studies operationalize “champion” as a dichotomous variable that is either present or absent, which does not enable rigorous examination of the mechanisms of a champion’s influence—positive or negative—on implementation outcomes; nor does it account for potential interactions between champions and their organizational context.

As a case example for better understanding champions, we examined implementation of recommended immediate postpartum contraceptive services. Champions appear to play a powerful role in implementing this evidence-based practice [14–16]. Immediate postpartum contraception involves the insertion of a long-acting reversible contraceptive (LARC; e.g., intrauterine device or contraceptive implant) during hospitalization for childbirth. Although national guidelines recommend universal access to this service, it is provided almost exclusively at a small number of “early adopter” academic medical centers [14, 17], and immediate postpartum LARC utilization remains rare [17, 18]. Implementation is complex, requiring coordination across settings (e.g., outpatient and inpatient) and often siloed stakeholder groups (e.g., providers, pharmacy, and billing) [15, 16]. It is unclear how implementation champions navigate these complexities and whether certain champions are more likely to succeed. As a case example, we examined inpatient postpartum LARC implementation within early adopter sites to identify and understand how attributes of champions influenced implementation outcomes.

## Methods

We conducted a national comparative case study of the implementation of inpatient postpartum contraceptive care at 11 U.S. maternity hospitals in 2017–18. We used a comparative, multiple case study design with a goal of analyzing similarities and differences across cases in order to produce generalizable knowledge about how and under what circumstances implementation unfolds successfully [19, 20]. We report our methods according to the Consolidated Criteria for Reporting Qualitative Research (COREQ) [21]. We selected COREQ (Additional file 1) because of its detailed focus on the collection, analysis, and reporting of interview data, such as that used in the current study. This study was deemed exempt human subjects research by the University of Michigan institutional review board (HUM00127245).

LARC service provision at the hospital level is difficult to identify within national administrative datasets. Thus, we conducted a systematic literature search in PubMed to find published literature related to research studies on immediate postpartum contraceptive care. Of 17 unique academic medical centers identified, nine were currently offering immediate postpartum LARC services and willing to participate. For each hospital, we first identified the “champion” (i.e., the individual leading implementation efforts) and invited them to participate in an initial telephone interview. We asked about their experiences with implementation, including potential organization and patient population characteristics that might have impeded or promoted implementation. We used snowball sampling with these champions to identify and recruit two additional hospitals that had very recently implemented services but had not conducted research trials of peripartum contraceptive care.

We then conducted single-day site visits, which included additional semi-structured interviews with key informants—defined as individuals with unique knowledge about implementation based on their role within the organization. We asked the site champion to select key informants with a goal of representing various groups’ perspectives in describing implementation (e.g., maternity attendings, nurses and residents, pharmacy staff, revenue staff, and hospital administrators) [22, 23]. Interviews were audio-recorded with permission and professionally transcribed verbatim. Rarely, when key informants were unavailable on the day of a study site visit, interviews were completed by telephone (*n* = 4) or email (*n* = 1).

Data collection (i.e., semi-structured interview guide) and analyses (i.e., coding framework and site summaries) were guided by the Consolidated Framework for Implementation Research (CFIR). CFIR domains include factors that may influence implementation, including the intervention itself (immediate postpartum contraception), the inner (clinic and hospital) and outer (payer policy, region, country) settings, the involved individuals, and the implementation process [24]. The interview guide contained items and probes about each CFIR construct, including formal implementation leaders. We did not ask about specific attributes of champions, but many interviewees spoke at length about their site’s champion(s), and key champion attributes thus emerged from the data during analysis. Specific items and probes in the interview guide were refined via pilot testing with our institution’s interdisciplinary program on Women’s Healthcare Effectiveness Research.

Consensus coding was used throughout our analysis. In our first analysis phase, qualitative content analysis using a priori codes, the CFIR was used to guide coding of qualitative data. Coded data were then assigned quantitative ratings indicating valence and strength of influence of each CFIR construct on implementation efforts [25]. The criteria used for assigning quantitative ratings are provided in Additional file 2. We catalogued which implementation strategies had been used by each site, guided by the 73 Expert Recommendations for Implementing Change (ERIC) strategies, but also allowing other implementation strategies to emerge [26]. Implementation success was defined qualitatively based on the extent to which inpatient contraceptive provision was routinized and sustainably integrated into standard maternity services with three observed levels: services were routinely available, services were made available but then de-implemented, and services were not routinely available.

**Fig. 1:**
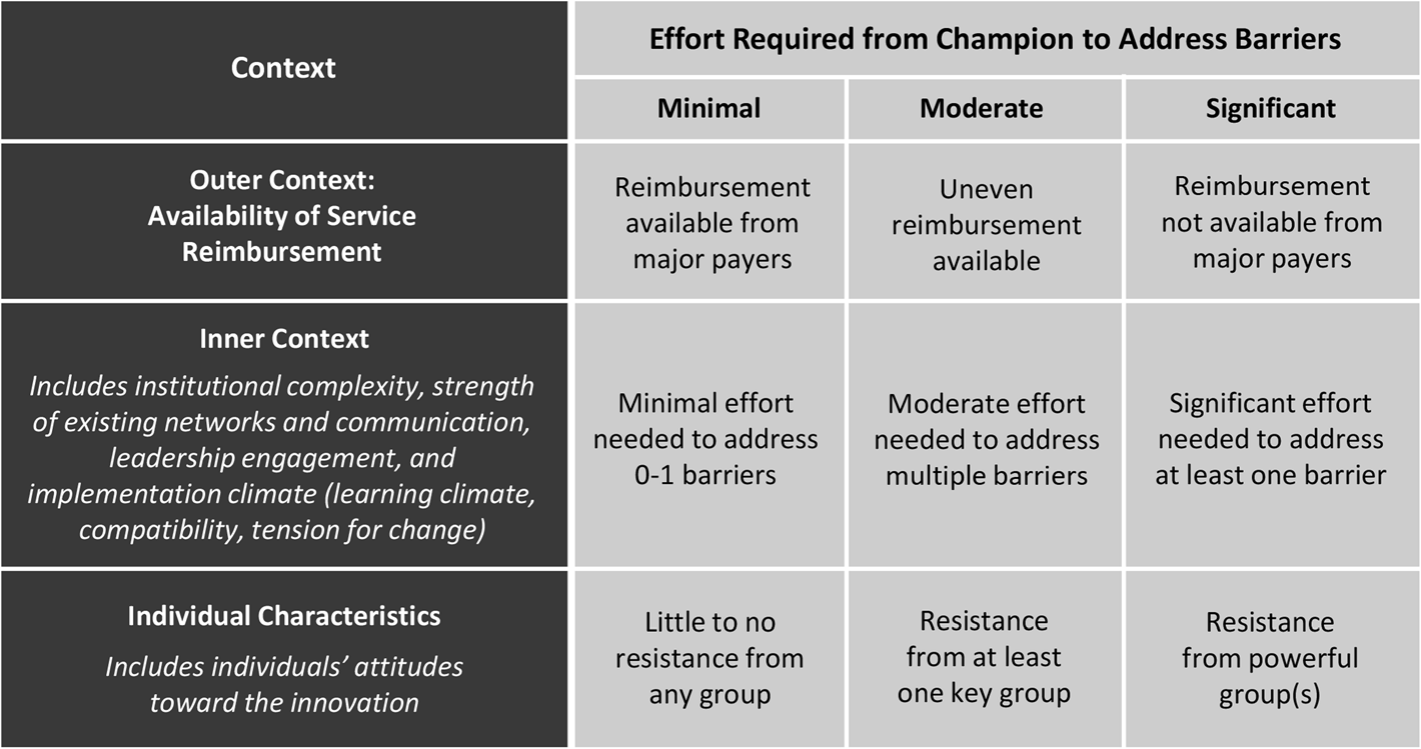
Qualitative criteria used to categorize context for implementation

The strong influence of champions on implementation outcomes emerged in our analyses. We also observed that implementation outcomes differed despite champions using relatively similar implementation strategies—suggesting that who a champion was, along with their context, might influence outcomes. Therefore, we conducted a deeper, inductive content analysis of champions for each site, exploring champion attributes that seemed to influence implementation. First, we identified and elaborated emerging attributes of champions, focusing on those linked by interviewees to outcomes. Second, we rated the demonstrated presence of champion attributes in each site. Third, to understand each champion’s local context for implementation, we categorized site barriers as requiring minimal, moderate, or significant effort from champions to avoid impeding implementation (Fig. 1). We focused on barriers posed by outer context, inner context, and individual characteristic domains, as constructs within these domains had emerged in phase one analysis as strongly affecting implementation outcomes (defined by + 2 or − 2 ratings in a majority of sites). Finally, we developed matrices of findings to support cross-case synthesis, identifying patterns of individual attributes, context, and implementation outcomes [27].

Our research team members were all female, including research assistants (KB, MW) and three physicians with training in qualitative research (VD, MH, MHM) and obstetrics and gynecology (VD, MHM), who were employed at the University of Michigan. Interviews were conducted by MW and MM. Coding was done by KB, MW, and MM, assisted by a master’s student (KA), using the Dedoose software. Consultation on qualitative research was provided by researchers with extensive experience in the field and deep familiarity with CFIR and the champion’s literature (LJD, JHF).

## Results

### Data collected

Participating hospitals (*n* = 11) were medium to large, with annual delivery volumes ranging from 2400 to 8500 births per year. We interviewed 78 key informants (73 in person, four by telephone, and one by email). Interview duration ranged from 11 to 65 min (mean 35 min), with 5–10 key informants (mean 7.1) per site. Interviewees included implementation champions (*n* = 12), front-line clinicians (*n* = 33), operations staff (*n* = 24), and hospital administrators (*n* = 9).

### Implementation outcomes and context

Nine of the 11 participating hospitals successfully and sustainably routinized services (Table 1). One site successfully routinized services but later de-implemented due to outer setting barriers. One site had not successfully routinized services.

**Table 1:**
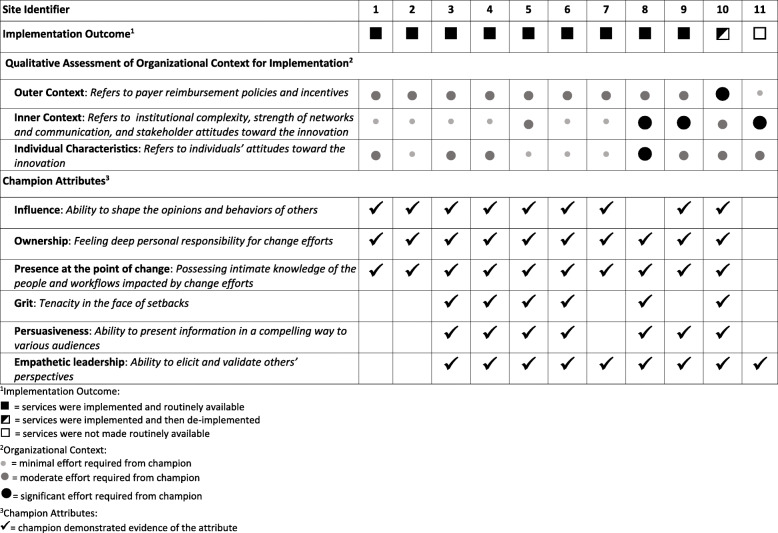
Implementation outcome, context for implementation, and champion attributes, by site

Across sites, champions worked within varied contexts, with differing degrees of barriers posed by the outer context, inner context, and individual characteristics. Outer context barriers were largely related to financial disincentives established by insurance payment policies for the new service. Inpatient delivery services are often reimbursed with a single, global payment that does not increase with the provision of inpatient LARC, despite the expense of these devices (approximately $800 each). Since 2012, some Medicaid agencies have begun providing reimbursement for inpatient LARC with a separate, unbundled payment, but site 10 faced a particularly unfavorable outer context in which payers had promised reimbursement for new services, but payments never materialized. Other sites experienced moderate (sites 5 and 10) and significant (sites 8, 9, and 11) inner context barriers to implementation success (e.g., challenges posed by organizational size and siloing, poor existing relationships and communication processes across stakeholders, poor learning climates, and workflow incompatibility). Site 11’s champion faced a particularly challenging inner context for implementation, with resistance to change from hospital administrators, in addition to other inner context barriers At this site, department leaders supported implementation of inpatient contraceptive implants, but had concerns about the safety of inpatient IUD provision and failed to reach consensus about whether providers should have separate clinical privileges for inserting IUD postpartum. Ultimately, without agreement from hospital administrators, this site was unable to offer inpatient IUDs.

### Key champion attributes

The champions leading implementation at study sites were all obstetrician-gynecologists. Some champions had additional fellowship training in family planning (*n* = 6) or maternal fetal medicine (MFM; *n* = 1). At one site (site 5), two physician champions with different expertise (one generalist obstetrician and one MFM) worked together to spearhead implementation. Most champions worked on the maternity unit and were familiar with its day-to-day care delivery operations. None had dedicated time allocated to implementation activities. Champions across sites used similar strategies to promote implementation, including developing stakeholder interrelationships, building a coalition to lead implementation, training and educating stakeholders, utilizing financial strategies, staging implementation scale-up, and changing infrastructure.

In the ensuing sections, we present qualitative evidence that six key attributes were associated with champions’ success at implementing new practices: (1) influence, (2) ownership, (3) physical presence at the point of change, (4) grit, (5) persuasiveness, and (6) participative leadership style. We describe how each attribute influenced a champion’s ability to overcome implementation barriers or leverage facilitators within their organization and thereby drive downstream implementation outcomes at their site.

#### Influence

Champions wielded varying degrees of influence to engage colleagues and overcome organizational barriers to change. Influence stemmed from three sources: formal authority, informal authority, and institutional savvy.

Six champions (sites 1, 2, 4, 5, 6, and 10) had formal authority from serving in highly visible leadership roles in their institution (e.g., division director, residency program director). At two sites (sites 1 and 2), champions had sufficient authority to launch implementation efforts without any additional permission from others:

"My approach is ‘ask forgiveness; not permission.’ So, we didn’t ask anybody if it’s okay if we do this. We just started doing it. And, oh, okay. No one really brought up any objections, so we just kept doing it." (Champion, Site 1)

Their formal authority afforded them entree into siloed segments of the organization and helped ensure responsiveness to their requests. At three sites (sites 3, 7, and 9), champions did not have formal authority themselves, but wielded that of others. For example, one champion diffused resistance by advertising a department chair’s support for the initiative:

"... when you can say, ‘Look, [Department Chair] thinks this is something we need to do and it’s important,’ that kind of empowers you to squash any sort of negativity about it. I mean, there are still barriers but, basically, it empowers you to kind of get through those barriers instead of be stopped by them, you know?" (Champion, site 9)

At all sites where champions had formal authority and at two additional sites (sites 3 and 9), interview data suggested that champions had informal authority. They were respected and implicitly trusted by colleagues, and many had a reputation as a leading expert in immediate postpartum contraception. Many had trained or had long tenure at the hospital. These champions readily shaped the attitudes and behaviors of others and could effortlessly engage colleagues in the change effort:

"… people buy into what she has to say very easily, and we trust her." (Attending, Site 9)

"... we just all knew that when [Champion] was excited about it, we should also be excited about it." (resident, site 4)

The third source of influence came through institutional savvy, where champions instinctively navigated the social architecture and culture of their organization to overcome stakeholder resistance (sites 2, 3, 4, 5, 6, 7, and 10). They understood who would likely oppose the initiative and leveraged personal relationships to engage those colleagues. One pharmacist described how her decade-long relationship with the site champion eclipsed her initial wariness about the initiative:

"… [for] me to go ahead and invest my time into a money losing proposition, it wasn’t really high on my radar. But, again, working with [Champion] for so many years, it just was like he had asked for it and we said, yes, we would do this for him." (Pharmacist, site 4)

One champion (site 8) with lack of influence struggled for years to implement services. She ultimately partnered with a senior project manager with informal authority and institutional savvy. The project manager helped the champion build relationships across silos, overcome resistance, and spur collective action in order to successfully implement new services. Another champion without influence failed to implement inpatient contraceptive services (site 11). She did not have formal authority and could not leverage power-by-proxy, because she faced resistance from hospital administrators. The Chief Medical Officer (CMO) had reservations about the safety of inpatient postpartum intrauterine devices (IUD); the champion tried multiple times, but ultimately failed to engage the CMO and the Department Chair:

“And it just kind of didn’t happen and I just never heard back from either of them and I felt kind of paralyzed where I couldn’t really move forward without knowing whether it was okay or not.” (Champion, site 11)

This champion had a relatively short tenure at the site and lacked the institutional savvy demonstrated by other influential champions. She was viewed as an expert about contraceptive care, but her influence remained limited due to resistance from more powerful opinion leaders like the CMO.

#### Ownership

Champions who took personal responsibility for the implementation initiative’s success prioritized and devoted the time needed to achieve implementation goals. At all successful sites, champions were intrinsically motivated and voluntarily took on leadership of implementation through self-initiative, rather than institutional mandate:

“… one of the things I decided I wanted to see happen here was for us to have a postpartum LARC program… and I think it was pretty much just a matter of me telling other people, ‘Is there some reason why we are not doing this?” (Champion, site 1)

Champions who took ownership strongly identified with their role and demonstrated commitment to leading implementation and facilitating behavior change in others. These champions often devoted an extraordinary amount of personal time to implementation activities. They described working nights and weekends, above and beyond their often demanding clinical responsibilities. Many provided 24-h access to their personal cell phone for questions from colleagues as service delivery began. Conversely, the champion at the site with failed implementation had been tasked to lead implementation by department leadership. This champion admitted that postpartum contraception implementation was not a personal priority; she had another administrative role about which she was more passionate:

“Because to be honest with you… my top priority wasn’t postpartum [long-acting reversible contraception]... I honestly think that somebody else needed to have been the champion for this, not because I didn’t want to do it or didn’t believe in it but I just – it just wasn’t going to happen, I think, if it was me.” (Champion, site 11)

Competing demands on her time, coupled with lack of ownership, made championing efforts infeasible. Having a designated champion without ownership also prevented other potential champions from emerging. Two other physicians at this hospital said they would have been willing to lead the initiative but were hesitant to step on the named champion’s toes.

#### Presence at the point of change

Many interviewees described the importance of the champion being embedded on the maternity unit. Champions physically present at the front lines of clinical service delivery understood the culture and daily workflow on the unit. Being embedded enabled champions to effectively integrate contraceptive services into existing care delivery processes and address emerging workflow challenges (i.e., optimizing compatibility). They could readily provide needed information and keep front-line clinicians engaged. One site champion described the importance of her and her colleague being embedded on the delivery unit:

“... that is such a key thing to this success, because you have somebody that’s physically there doing it, seeing the day in/day out challenges for service provision in this setting, and that just helped us tremendously… they know everybody, and they were able to really get people on board...” (Attending, site 10)

Additionally, the champion’s presence on the delivery unit made the initiative visible and helped hold colleagues accountable for executing new workflows (e.g., using new order sets or electronic documentation elements). By being present at the point of change, champions communicated “I’m here if you need me,” as well as “I’m watching you.” One champion did not work on the delivery unit often, but recognizing the value of a more constant presence on the unit, she recruited a generalist attending to co-lead the implementation initiative with her.

At the site that had not institutionalized services, the champion was a family planning attending who did not work on the maternity unit. Interviewees recognized that not having an embedded champion was a missed opportunity to heighten the relative priority and visibility of the initiative, citing this as a reason the initiative had “fallen by the wayside” (Hospital administrator, site 11).

#### Grit

Champions with grit had tenacity and resilience that enabled them to overcome setbacks. At six sites (sites 3, 4, 5, 6, 8, and 10), champions demonstrated grit when experiencing multiple, recurring barriers. One champion, for example, described the effort required to convince her pharmacy colleagues to stock contraceptive devices for postpartum provision, underscoring how her persistence ultimately overcame resistance:

“It was incredibly tedious and painful and we still ended up with a lot of… barriers that ended up being like, ‘well, where are the devices going to be stored? Well, can't they be stored in the pharmacy and be sent up? Well… maybe… Well, there’s not room on labor and delivery. Well, can we get a new Pyxis? Well, I don't know and I don't know if there’s room to place it there...’ And then, the stuff came up again about how people lost money by placing them inpatient rather than outpatient… We did like a test case where—I got permission to take a device from clinic and put it in a postpartum patient and bill for it... just to see if they would actually pay it... So then, based on that, we created this big budget that had to go to the pharmacy committee and like hospital committee to approve it for the pharmacy, and they denied it… they wanted it restricted to like maybe only two people in the hospital could actually order the devices, which was just not practical. But we ended up coming up with a lot of different things and then they finally approved it...” (Champion, site 8)

Multiple sites shared similar stories of recurring institutional barriers, which took continued persistence to resolve. Champions with grit responded to barrier after barrier with energy, nimbleness, and resourcefulness. These champions were described as relentlessly undeterred:

“... [Champion] had to fight a lot of battles… at every level in terms of the level of billing, the level of pharmacy, you know, all of the details you don’t really think about when you are starting a program like this, like, she really had to iron out… she just wanted to get it done.” (Resident, site 8)

At the site where services were not routinized, the champion felt overwhelmed by the complexity and amount of work demanded by the initiative. She was perceived by peers as being too fatigued to intensify her efforts and overcome setbacks:

“There are definitely people who are aware and who have been trying to do this for a while… And I'm sure – I think some of them also just feel like when you ask for something repeatedly and you continue to try – and try and try and try for years to put this into place and it feels like you are not getting anywhere, I would imagine some level of fatigue sets in. Like, how many times can you ask and jump through the hoops?” (Attending, site 11)

#### Persuasiveness

Many champions were highly skilled communicators. They were described as inspiring and able to convey authentic enthusiasm for the initiative to various groups in the organization. Their persuasiveness stemmed from a genuine, deep-rooted belief in the merits of the new service. They exuded a “contagious” passion that helped colleagues understand why inpatient contraceptive care was important for patients and created tension for change:

“... a lot of that is from [Champion’s] passion for this and, you know, ability to kind of go out there and rally the troops and get everyone behind it… She’s a good salesperson, like, she can but she backs it up with data you know? She’s like, ‘This is what I want to do and this is why I think we should do it and we want to be ahead of the game… people are starting to do it across the country, let’s get on board now’… and when she presents and talks about it you can tell how passionate she is about it.” (Pharmacist, site 6)

Persuasive champions understood the importance of tailoring messages to maximally engage various stakeholders and anticipated colleagues’ knowledge gaps and concerns and responded in ways that resonated. For example, persuasive champions often described using evidence from scientific literature to engage physicians, focusing on patient needs and protocols when engaging nurses, and discussing projected financial outcomes when engaging pharmacy staff. Tailored persuasiveness helped champions meet the unique informational needs of different groups.

Persuasive champions targeted communication efforts toward resistors with the most power to impede implementation. For example, these champions often devoted significant effort to combating resistance from pharmacy staff who were the “device gatekeepers,” as one champion called them, recognizing that they could easily halt operations if not engaged. Conversely, site 11 demonstrates how a failure to persuade powerful stakeholders, including hospital administration, which was ruinous for implementation efforts.

#### Participative leadership style

Effective champions were often described as having a participative leadership style that facilitated collective action. Such champions involved their colleagues in decision-making about how to embed the new practice within existing care delivery and welcomed ongoing feedback. This helped them design workflows to meet the preferences of front-line clinicians and refine implementation in real time:

“And so, you are going to the lead team meetings and the midwife meetings, checking with nurses, kind of going back with people to see, you know, what worked, what didn’t, when this one fell through the cracks, where were you looking where you thought you had the right information and didn’t” (Champion, site 10)

Champions with a participative leadership style created a learning climate in which others felt included, heard, and important. They demonstrated a persistent curiosity about the perspectives of colleagues affected by the change and listened with the same enthusiasm with which they would want to be heard. Colleagues responded, and were motivated to actively participate in implementation, because they felt like essential and valued contributors to the change process:

“[Champion] was very respectful and came and educated everybody about it and heard their concerns and what, you know, they thought would be barriers before we actually, like, rolled it out, and I think that helped that the nurses felt that they were part of the process.” (Attending, site 3)

Guided by a commitment to leaving no colleague behind, participative leaders used empathy to overcome fears about the change effort. They anticipated, for example, that clinicians might feel vulnerable when offering a new procedure and provided a needed confidence boost:

“... it’s helpful to have people come and be just completely confident and, like, ‘of course you can do this’ and... ‘you do things way harder than this’” (Midwife, site 2)

One champion shared a story about how she responded to nurses’ apprehension about answering patient questions about the new service. She provided language to use at the bedside and emphasized that the initiative required everyone’s collective effort, reminding them “you guys have a big role, like, we cannot do this without you and we need you guys to be on board and be passionate” (Champion, site 6). By helping colleagues feel safe voicing concerns and feel like a valued contributor to the change effort, participative leaders could meaningfully engage colleagues and design systems where desired behavior change was easy and sustained.

A participative leadership style was not required for success, however, as demonstrated by site 1. In contrast to the participative leadership observed in other champions, this site’s champion demonstrated relative indifference to colleagues’ perspectives about the new practice:

“...we have one staff member who is a very devoted breastfeeding lobbyist, and I think she raised concerns. But I think that it’s just like yeah, okay, sorry. You know, we don’t see that there is a problem here. The data don’t indicate a problem. The WHO and the CDC say there’s not a problem…” (Champion, site 1)

Despite this champion’s style, services were still routinized at site 1—perhaps because the champion allowed the new service to passively diffuse from a handful of early adopters to others in the organization, rather than launching a formal implementation effort. Participative leadership was also not sufficient for implementation. Site 11 did not routinize services, despite having a champion who demonstrated participative leadership and effectively engaged clinicians, pharmacists, and billing staff.

## Discussion

In this comparative case study, we identified six key champion attributes as potential facilitators of implementation. We observed that a complex interplay between champion attributes and context contributed to implementation outcomes, rather than the distinct presence or absence of a champion. Our findings help elucidate how and why champions work, providing qualitative evidence that champion attributes may affect outcomes by influencing their ability to navigate implementation barriers within their organization—especially related to structural characteristics, networks and communications, implementation climate (tension for change, learning climate, perceived compatibility of new practices with existing workflow and norms and values), and stakeholder attitudes toward the innovation.

The existing literature has generally operationalized the concept of champions as a dichotomous state: did the initiative have a champion or not? Our findings affirm the call by others [1] for a more nuanced conceptualization of the important role of champions: to what degree did champion(s) have the key attributes outlined by our findings and how well were they aligned with local contextual barriers? It is important to assess the nature of contextual barriers to implementation and identify or groom champions who are well equipped to address them. Change efforts may benefit from champions who can leverage organizational influence, or power-by-proxy, and from teams with champions embedded at each point of change—particularly if resistance is anticipated. Organizations may also consider allowing both leaders and team members to emerge as champions (rather than being tasked as a champion), if possible, as this may promote more ownership for the initiative. Persuasiveness, grit, and participative leadership may be teachable skills, while influence can be strengthened by conferring formal authority and possibly by building opinion leadership and institutional savvy. This suggests that champions can be developed with the right support. Cultivating these characteristics, especially in more than one person, may help ensure successful implementation and better prepare improvement teams for future initiatives. Importantly, some outer context barriers are likely outside the sphere of influence of organizational champions; this is one situation in which champions may be necessary but insufficient.

Our findings should be interpreted in light of our design’s limitations. This was a retrospective, comparative case study that relied on participant recall, and findings about the attributes of effective champions, their teams, and potential mechanisms of action are hypothesis-generating. From our small sample, we cannot say which champion attributes are necessary or sufficient, nor can we isolate the unique effects of individual attributes. However, the contextual variation across sites and robust qualitative methodology provide rich, new understanding about how certain attributes may help champions navigate implementation barriers. Future work should prospectively evaluate potential causal pathways between champion attributes, the implementation strategies utilized, and implementation outcomes. All champions interviewed for this study were physicians. It may be beneficial for future work to examine whether professional role moderates the effects of implementation champions. Finally, participating hospitals were early adopters of immediate postpartum contraceptive care, and most were successful. Future evaluation including a larger number of sites without full success may better elucidate attributes that contribute to champion ineffectiveness.

## Conclusions

The importance of champions for healthcare change efforts is well established. Our findings highlight the key role of specific champion attributes: their success does not lie just in *what* they do, but also in *who* they are. Influence, ownership, physical presence at the point of change, persuasiveness, grit, and participative leadership may all contribute to a champion’s ability to drive implementation outcomes. Our findings can be used to select and groom more effective champions for change efforts in healthcare.

## Supplementary information

**Additional file 1.** Completed COREQ Checklist.

**Additional file 2.** Table. Criteria for assigning quantitative ratings to CFIR constructs.

## Data Availability

Some data generated or analyzed during this study are included in this published article. Additional data (generated and analyzed) are available from the corresponding author on reasonable request.

## Abbreviations

CFIR: Consolidated Framework for Implementation Research
LARC: long-acting reversible contraceptive
ERIC: Expert Recommendations for Implementing Change
MFM: Maternal fetal medicine
CMO: Chief Medical Officer
IUD: Intrauterine device

## References

[1] Miech EJ, Rattray NA, Flanagan ME, Damschroder L, Schmid AA, Damush TM (2018). Inside help: an integrative review of champions in healthcare-related implementation. SAGE Open Med..

[2] Schon DA (1963). Champions for radical new inventions. Harv Bus Rev..

[3] Damschroder LJ, Banaszak-Holl J, Kowalski CP, Forman J, Saint S, Krein SL (2009). The role of the champion in infection prevention: results from a multisite qualitative study. Qual Saf Health Care..

[4] Clack L, Zingg W, Saint S, Casillas A, Touveneau S, da Liberdade JF (2018). Implementing infection prevention practices across European hospitals: an in-depth qualitative assessment. BMJ Qual Saf..

[5] Krein SL, Damschroder LJ, Kowalski CP, Forman J, Hofer TP, Saint S (2010). The influence of organizational context on quality improvement and patient safety efforts in infection prevention: a multi-center qualitative study. Soc Sci Med..

[6] Saint S, Kowalski CP, Banaszak-Holl J, Forman J, Damschroder L, Krein SL (2010). The importance of leadership in preventing healthcare-associated infection: results of a multisite qualitative study. Infect Control Hosp Epidemiol..

[7] Flanagan ME, Plue L, Miller KK, Schmid AA, Myers L, Graham G (2018). A qualitative study of clinical champions in context: clinical champions across three levels of acute care. SAGE Open Med..

[8] Crabtree BF, Miller WL, Tallia AF, Cohen DJ, DiCicco-Bloom B, McIlvain HE (2005). Delivery of clinical preventive services in family medicine offices. Annals of family medicine..

[9] Soo S, Berta W, Baker GR. Role of champions in the implementation of patient safety practice change. Healthc Q. 2009;12 Spec No Patient:123-8. 10.12927/hcq.2009.20979 19667789

[10] McCabe MP, Karantzas GC, Mrkic D, Mellor D, Davison TE (2013). A randomized control trial to evaluate the beyondblue depression training program: does it lead to better recognition of depression?. Int J Geriatr Psychiatry..

[11] Acolet D, Allen E, Houston R, Wilkinson AR, Costeloe K, Elbourne D (2011). Improvement in neonatal intensive care unit care: a cluster randomised controlled trial of active dissemination of information. Arch Dis Child Fetal Neonatal Ed..

[12] Naylor PJ, Macdonald HM, Zebedee JA, Reed KE, McKay HA (2006). Lessons learned from action schools! BC--an 'active school' model to promote physical activity in elementary schools. J Sci Med Sport..

[13] Slaunwhite JM, Smith SM, Fleming MT, Strang R, Lockhart C (2009). Increasing vaccination rates among health care workers using unit "champions" as a motivator. Can J Infect Control..

[14] Okoroh EM, Kane DJ, Gee RE, Kieltyka L, Frederiksen BN, Baca KM, et al. Policy change is not enough: engaging provider champions on immediate postpartum contraception. Am J Obstet Gynecol. 2018;218(6):590 e1- e7. 10.1016/j.ajog.2018.03.007 PMC5970075 29530670

[15] Palm HC, Degnan JH (2020). Biefeld SD.

[16] Hofler LG, Cordes S, Cwiak CA, Goedken P, Jamieson DJ, Kottke M (2017). Implementing immediate postpartum long-acting reversible contraception programs. Obstet Gynecol..

[17] Moniz MH, Chang T, Heisler M, Admon L, Gebremariam A, Dalton VK (2017). Inpatient postpartum long-acting reversible contraception and sterilization in the United States, 2008-2013. Obstet Gynecol..

[18] Moniz MH, Soliman AB, Kolenic GE (2019). Tilea A.

[19] Goodrick D. Comparative case studies: methodological briefs-impact evaluation No. 9 2014 []. Available from: https://www.unicef-irc.org/publications/754-comparative-case-studies-methodological-briefs-impact-evaluation-no-9.html.

[20] Baxter P, Jack S (2008). Qualitative case study methodology: study design and implementation for novice researchers. The Qualitative Report..

[21] Tong A, Sainsbury P, Craig J (2007). Consolidated criteria for reporting qualitative research (COREQ): a 32-item checklist for interviews and focus groups. Int J Qual Health Care..

[22] Patton M (2002). Qualitative Research and evaluation methods.

[23] Kuper A, Lingard L, Levinson W (2008). Critically appraising qualitative research. BMJ..

[24] Damschroder LJ, Aron DC, Keith RE, Kirsh SR, Alexander JA, Lowery JC (2009). Fostering implementation of health services research findings into practice: a consolidated framework for advancing implementation science. Implement Sci..

[25] Damschroder LJ, Lowery JC (2013). Evaluation of a large-scale weight management program using the consolidated framework for implementation research (CFIR). Implement Sci..

[26] Powell BJ, Waltz TJ, Chinman MJ, Damschroder LJ, Smith JL, Matthieu MM (2015). A refined compilation of implementation strategies: results from the Expert Recommendations for Implementing Change (ERIC) project. Implement Sci..

[27] Miles MB, Huberman AM (1994). Qualitative data analysis: an expanded sourcebook.

